# Tetratricopeptide repeat domain 9A knockout induces social anxiety and impairs offense behaviors in female mice

**DOI:** 10.22038/IJBMS.2022.63044.13932

**Published:** 2022-06

**Authors:** Wing Shan Yu, Li Guan, Shawn Zheng Kai Tan, Smeeta Shrestha, Yu Zuan Or, Thomas Lufkin, Valerie CL Lin, Lee Wei Lim

**Affiliations:** 1 Neuromodulation Laboratory, School of Biomedical Sciences, Li Ka Shing Faculty of Medicine, The University of Hong Kong, Hong Kong SAR, P.R. China; 2 Department of Physiology, Guangzhou University of Chinese Medicine, Guangdong, P.R. China; 3 School of Biological Sciences, Nanyang Technological University, 50 Nanyang Avenue, Singapore; 4 School of Basic and Applied Sciences, Dayananda Sagar University, Bangalore, India; 5 Department of Biology, Clarkson University, Potsdam, New York, United States

**Keywords:** Aggression, Anxiety, Behavioral tests, Social behavior, Tetratricopeptide repeat - domain 9A (TTC9A)

## Abstract

**Objective(s):**

The involvement of tetratricopeptide repeat domain 9A (TTC9A) in anxiety-like behaviors through estrogen action has been reported in female mice, this study further investigated its effects on social anxiety and aggressive behaviors.

**Materials and sMethods:**

Using female Ttc9a knockout (*Ttc9a-/-*) mice, the role of TTC9A in anxiety was investigated in non-social and social environments through home-cage emergence and social interaction tests, respectively, whereas aggressive behaviors were examined under the female intruder test.

**Results:**

We observed significant social behavioral deficits with pronounced social and non-social anxiogenic phenotypes in female *Ttc9a-/-* mice. When tested for aggressive-like behaviors, we found a reduction in offense in *Ttc9a-/-* animals, suggesting that TTC9A deficiency impairs the offense responses in female mice.

**Conclusion:**

Future study investigating mechanisms underlying the social anxiety-like behavioral changes in *Ttc9a-/-* mice may promote the understanding of social and anxiety disorders.

## Introduction

Social well-being is of vital importance for the quality of life and survival; loneliness and weaker social relationships have been associated with various medical conditions and mortality risk (1-3). Anxiety disorders are among the most prevalent threats to mental health worldwide (4) and have been linked with social impairment (5). Patients with anxiety disorder have a significantly increased trend of social dysfunction, and social disability is predictive of persistence of anxiety, with social impairment remaining even after remittance of anxiety disorder/depression (6, 7). Although anxiety disorders are associated with social withdrawal, individuals suffering from anxiety often present excessive aggression that could further worsen social and even physical health (8-10). Understanding the neurobiological connections between anxiety and impaired social functioning is important to developing more efficient therapeutic strategies. 

The tetratricopeptide repeat domain 9 (TTC9) is a protein family containing tetratricopeptide repeat (TPR) domains constituted of the 34 amino acid consensus motif present in various number of tandem repeats (11). The TPR motifs are generally arranged in antiparallel α-helical hairpins which are clustered to form flexible grooved interfaces for protein interactions involved in a diverse range of biological functions, including cell cycle control, transcription, protein folding, and steroid receptor signaling (11-13). Among the TTC9 family, TTC9A has garnered attention for its role in anxiety-like behaviors in female mice (14). TTC9A is extensively expressed in neural plate tissues during mouse embryonic development day 13.5 (14). In humans, TTC9A is widely expressed in various tissues, with the highest expression in the brain (15, 16). TTC9A has been shown to both regulate (17) and be regulated (15, 16) by estrogen, a hormone heavily involved in mood regulation (18). The interactive effects between TTC9A and estrogen have been suggested to contribute to anxiety in female mice through mechanisms involving serotonergic functioning and neuroplasticity changes (14, 19). 

Despite involvement of TTC9A in anxiogenic behaviors, and the links between anxiety and social impairment (5, 6, 14), the role of TTC9A in social functioning, including social anxiety and aggressive-related responses, has not been well investigated. In this study, we probed the role of TTC9A in anxiety within non-social and social contexts, and in aggressive-like behaviors in a *Ttc9a* knockout (*Ttc9a*^-/-^) female mouse model using a battery of behavioral tests. 

## Materials and Methods

***Subjects***

Female *Ttc9a*^-/-^ (n=6) and wildtype C57BL/6J mice (WT; n=7) aged 5 months were bred in the Animal Research Facility, Nanyang Technological University, Singapore. *Ttc9a*^-/-^ mice were generated through a functional deletion of the *Ttc9a* gene by homologous recombination (14, 17). In short, the vector targeting *Ttc9a* was generated by substituting the *Ttc9a *exon 1 with a loxp flanked neomycin cassette, and was electroporated into R1 embryonic stem (ES) cells. Transfected ES cells were screened for homologous recombinant by Southern blotting, and cells with heterologous integration of the target vector were microinjected into 8 cells stage C57BL/6J embryos. The resulting *Ttc9a* chimeras heterozygous for *Ttc9a* exon 1 deletion were bred with C57BL/6J wildtype mice to generate *Ttc9a *heterozygous mice. These heterozygous mice were crossed to generate the *Ttc9a*^-/-^ and *Ttc9a*^+/+^ mice. Mice were genotyped by PCR analysis of tail DNA using the following primer pairs: wildtype: forward 5’-GAG CGA TCG CAG GAG GAG-3’, reverse 5’- CCA AGC CCT TCC TCT CCA-3’; mutant: forward 5’-GAG CGA TCG CAG GAG GAG-3’, reverse 5’- CCA GAC TGC CTT GGG AAA AG-3’. The mice were socially housed in groups of 3–4 in Tecniplast individually ventilated cages with standard corncob bedding materials in a controlled room environment (24 °C–26 °C cage temperature, 60%–70% humidity, 12/12 hr light/dark cycle). Mice were given access to food and water *ad libitum*. All experimental procedures were approved by the Institutional Animal Care and Use Committee, Nanyang Technological University, Singapore (reference number: ARF-SBS/NIE-A0169 AZ).

***Experimental design***

**Figure 1:**
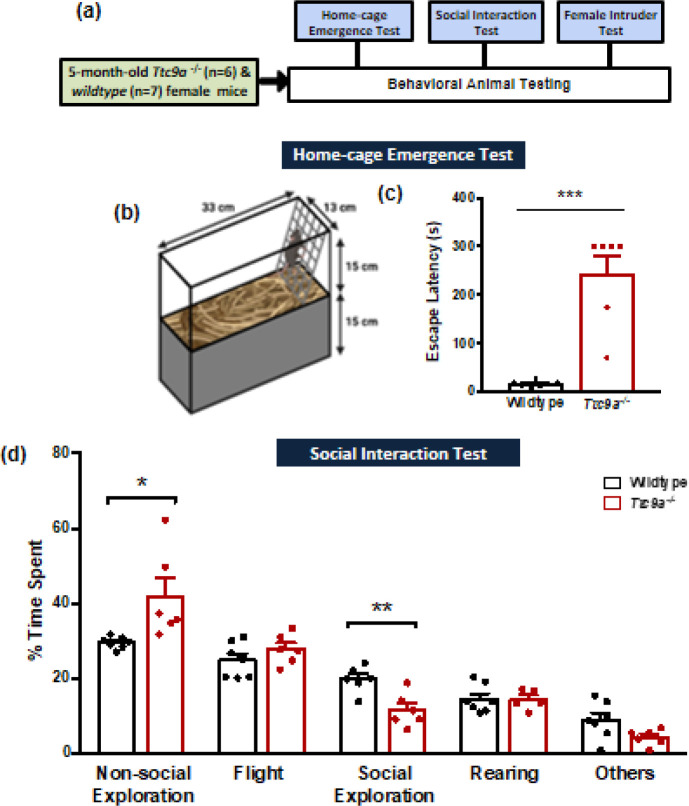
*Ttc9a-/- *female mice show anxiogenic-like behavior and deficits in social behavior. (a) A schematic diagram represents the timeline of the experimental design. (b) Graphical representation of the home-cage apparatus used in the home-cage emergence test. (c) *Ttc9a-/-* female mice showed anxiogenic-like behavior with a significantly increased escape latency in the home-cage emergence test. (d) *Ttc9a-/- *female mice showed deficits in the social interaction test, with increased non-social exploration and decreased social exploration. Behavioral categories of which both groups showed less than 5% total time are collapsed into the ‘Others’ sector, including move towards, move away, freeze, and defensive upright posture. Data are presented as mean + SEM, * represents *P*<0.05, ** represents *P*≤0.01, *** represents *P*≤0.001, independent samples t-test or Mann Whitney *U*-test as appropriate

A single batch of 5-month-old *Ttc9a-/-* mice (n=6) and corresponding WT mice (n=7) were subjected to a battery of behavioral tests. Home-cage emergence test was performed to measure anxiety levels (14, 20), while social interaction and female intruder tests were used to investigate social behaviors (14, 21). The tests were conducted in the order listed which started from the least stressful to the most stressful test, to minimize the influence from the previous task on behavioral performance. The estimated sample size and the total number of animals were justified based on our previous experience and literature review (22). The power calculation predicts a significant effect at 40% with a standard deviation of 25%, and the significance level was set to 0.05 and power of 0.8. For the behavioral study, the number of animals in each group was determined using the following formula: N = 2(0.25)^2^(1.96+0.842)^2^/(0.40)^2 ^= 6.13 (n=6 animals) (22). In this study, all animals were included in the behavioral analysis regardless of the estrous cycle stages, as studies have shown that staging the behavioral results by phases of the estrous cycle offered no reduction in variability in behavioral tests (23-25). The timeline for the behavioral test battery is shown in Fig. 1a. 

***Behavioral testing procedures***

All behavioral studies and analyses were conducted by researchers blinded to the mice genotype and testing conditions. To avoid unnecessary stress during behavioral experiments, all tests were at least one day apart, and mice were handled by picking up from the tail and placed on the palm of the researcher for 5 min a day for 4 days before testing. All tests were performed during the dark phase under dim light conditions. Mice were allowed 30 min acclimatization period in the behavioral test room before testing. After each test, all set-ups were cleaned with 70% ethanol and air-dried before the next mouse was tested (14). All tests were recorded using a video camera and analyzed with the ANY-maze Behavioral Tracking Software (Stoelting Co, USA). The behavioral parameters were scored by an experienced researcher and later confirmed by another researcher, both were blinded to the subjects’ genotype.

Home-cage emergence test was conducted as previously described (14, 20). In brief, animals were placed in a home-cage (13 x 15 x 33 cm) with the lid removed (Fig 1b). A grid was placed over the edge of the cage to facilitate escape from the home-cage, and escape latency was recorded. The anxiogenic-like response is indicated by an increased latency to escape. The session was ended if the mouse did not escape from the home-cage within 5 min and a score of 300 s was given.

The social interaction test was conducted in a new mouse cage (13 x 15 x 33 cm) that was unfamiliar to the subjects before acclimation (21). At the end of the 30-min acclimation, the test subject and four 5-month-old female genotype-matched stranger mice were introduced at the time testing cage. For behavioral investigation, the time spent on different behavioral parameters was analyzed including social exploration (sniffing another’s body including the anogenital area), rearing (vertical exploration by standing on hind-limbs), move towards (moving towards another), flight (running away from another after an agonistic interaction), lateral threat (lateral posture towards another mouse, accompanied with an arched back and head directed to the floor or the opponent), chase (rapidly pursuing another), move away (moving away from another), freezing (prompt and rigid pausing with head up for at least 1 s), defensive upright posture (motionless standing on the hind legs against the opponent), and non-social exploration (exploring the arena, i.e. sniffing beddings, walls, etc.). The behavioral parameters recorded for each subject covered 300s of the testing period to ensure a standardized and unbiased analysis of the results. 

The female intruder test was an adapted paradigm of the male resident-intruder test following a previously established protocol (25). Animals were individually housed for 2 days before the test. The test started when an unfamiliar 5-month-old genotype-matched female intruder mouse was introduced into the resident cage and videotaped. For behavioral analysis, we recorded the attack latency which is the time between the introduction of the intruder and the first clinch attack, the total offense score which is the sum of the time spent on lateral threat, upright posture, clinch attack, keep down (pushing the intruder against the ground on its back), and chase, as well as social exploration, move towards, rearing, flight, rest and inactivity (sitting or hunching over), and non-social exploration. Similar to the analysis of the social interaction test, the total time spent on various behavioral parameters in each mouse should add up to 300s to facilitate a standardized analysis of the results. 

***Statistical analysis***

All data were analyzed using IBM SPSS Statistics 27. Behavioral data were analyzed using independent samples *t*-test with Welch’s correction. When normality was violated as assessed by Shapiro-Wilk’s test, Mann-Whitney *U*-test was performed. For social interaction and female intruder tests, behavioral parameters of which both groups reached less than 5% of the total time were categorized as ‘others’. Outliers were identified and discarded based on box-plot diagrams. Values above the upper quartile or below the lower quartile by 1.5 times the interquartile range were considered outliers. The figures represent mean + *SEM*. Significance was defined as *P*<0.05.

## Results

***Ttc9a*** ^-/-^ ***mice exhibited anxiety and social behavior deficits***

In the home-cage emergence test, *Ttc9a*^-/-^ mice showed a significant increase in latency to escape from the home-cage compared with wild-type animals (*U*=0, *P*=0.001; Figure 1c), indicating an increase in anxiogenic response. In the social interaction test, the major components of social behaviors in wildtype and *Ttc9a*^-/-^ mice include non-social exploration, flight, social exploration, and rearing. The percentage of time spent on various behavioral parameters was compared between wildtype and *Ttc9a*^-/-^ mice. Independent samples t-test revealed a significant increase in non-social exploration (*t*_(5.51)_ = 2.565, *P*=0.049; Figure 1d) and a decrease in social exploration (*t*_(10)_ = -3.611, *P*=0.005) in *Ttc9a*^-/-^ mice. No significant difference between genotypes was detected in rearing and flight (*t*_(10)_ = -0.116, *P*=0.91 and *t*_(11)_ = 1.208, *P*=0.252, respectively.) Overall results suggest a reduction in social behaviors in *Ttc9a*^-/-^ animals. 

***TTC9A*** ^-/-^ ***mice exhibited an impairment in offense behaviors***

**Figure 2:**
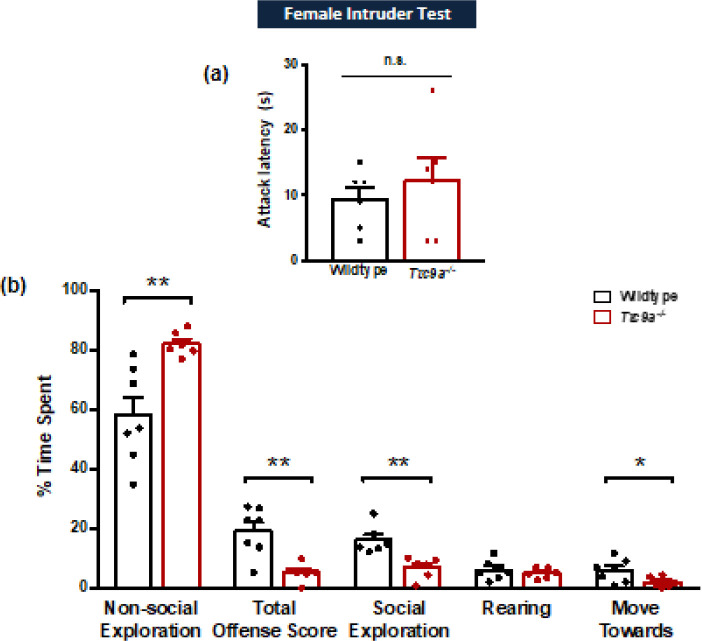
*Ttc9a-/-* female mice show impaired offense behaviors compared with wild-type. (a) *Ttc9a-/-* female mice showed no significant difference in attack latency in the female intruder test compared with wild-type mice. (b) *Ttc9a-/-* female mice showed an increase in non-social exploration and decrease in total offense score, social exploration, and move towards behaviors. Data are presented as mean + SEM, * represents *P*<0.05, ** represents *P*≤0.01, independent samples t-test or Mann Whitney* U*-test as appropriate

To examine if *Ttc9a* knockout had any effect on aggressive-like behaviors, the female intruder test was conducted. Independent samples t-test of attack latency showed no significant differences between genotypes (*t*_(10)_ = 0.711, *P*=0.493; Figure 2a). Among the behavioral parameters measured, wildtype and *Ttc9a*^-/-^ mice spent most of the time on non-social exploration. The composition of the other major behaviors includes total offense score, social exploration, rearing, and move towards. Comparisons of the time spent on various behavioral measures between wildtype and *Ttc9a*^-/-^ mice revealed a significant increase in non-social exploration in *Ttc9a*^-/-^ mice (*t*_(6.879)_ = 3.801, *P*=0.007; Figure 2b). Additionally, *Ttc9a*^-/-^ mice showed a reduced time spent on total offense score (*t*_(7.965)_ = -4.255, *P*=0.003), social exploration (*U*=36, *P*=0.002), and move towards behaviors (*t*_(11)_ = -2.264, *P*=0.045). Overall, the results suggest that *Ttc9a* knockout decreased offense behaviors and increased social avoidance in mice. 

## Discussion

In this study, we tested the anxiety-related behaviors in *Ttc9a*^-/- ^mice under both non-social and social settings using the home-cage emergence and social interaction tests, respectively. We validated our previous findings (14), showing increased anxiogenic responses in *Ttc9a*^-/- ^female animals in the home-cage emergence test. The home-cage emergence test is a test for neophobia that measures the tendency of mice to stay in a familiar environment (26). Depending on the neophobic nature of rodents, anxiety-like behavior is reflected by the avoidance of mice to exit their home-cage. Behavioral testing conducted in the familiar home cage environment offers an advantage of minimizing the stress that could mask behavioral differences between the wildtype and transgenic mice. We further looked into the social aspect of anxiety by conducting social interaction tests. Social interaction has been constantly tested as a measure of anxiogenic behavior in rodents, with the observation that anxiety-like states inversely impact the sociability of a rodent, while anxiolytics promote social interaction (21, 27-30). We extended our findings by showing social anxiety-like behaviors in the social interaction test indexed by a significant drop in social exploration (21). The reduction in social exploration was accompanied by a significant increase in non-social exploration and not by other tested parameters such as rearing or immobility. It provides evidence that the decreased social interaction in *Ttc9a*^-/- ^mice was not due to changes in locomotor activity. Consistently, *Ttc9a*^-/- ^mice spent significantly more time on non-social behavior and less time on social interaction during the female intruder test, which further indicates a sign of social withdrawal that is a typical symptom of social anxiety (31). 

With the common co-occurrence of anxiety with excessive aggression (8-10), it is interesting to examine the aggressive behaviors in *Ttc9a*^-/- ^female mice, which presented enhanced anxiogenic responses. The female intruder test adopted was modified from the male resident intruder test and has been validated in female rodent models of aggression induced by social isolation (25, 32, 33). Aggressive behavior in rodents is reflected by the clinch attack latency which measures the readiness to attack, whereas the total offense score is employed as an index of the intensity of aggression (34). Although no significant difference was found in the attack latency, there was a reduction in offense responses in *Ttc9a*^-/- ^mice during the female intruder test. Taken together, *Ttc9a*^-/- ^female mice exhibited an increase in social and non-social anxiety accompanied by a decrease in offense behaviors. Although the comorbidity of anxiety and aggression is often observed in human patients, they are not always co-regulated. For example, anxiolytics have been shown in both clinical and preclinical studies to reduce aggressive behaviors in some individuals while triggering aggression in others (10, 35). Furthermore, while the reduction in offense behaviors could be a direct result of *Ttc9a *knockout, we cannot rule out that it might be attributed to the social avoidance shown in *Ttc9a*^-/- ^mice.

We have previously demonstrated an increased anxiety level in ovariectomized* Ttc9a*^-/-^ female mice in response to estrogen treatment, suggesting that functional interaction between TTC9A and estrogen is crucial for its modulation of anxiety-like behaviors (14). It is perhaps unsurprising then the present study further shows impaired social behaviors in* Ttc9a*^-/-^ mice given estrogen role in it. In mice, knockout of estrogen receptor alpha (ERα) or beta (ERβ) leads to impaired social discrimination (36), while depletion of estrogen through ovariectomy also results in a decline in social memory and investigation (37). Similarly, women in the menopausal period have a higher incidence of social isolation due to estrogen deficiency (38, 39). Interestingly, 3 months of estrogen treatment in postmenopausal women led to increased anxiety in the social stress test, suggesting the effects of estrogen on social mood regulation may not be linear (40). These findings collectively indicate that changes in estrogen activity patterns, either increase or decrease, may result in social disturbances. The effects of estrogen on aggressive behaviors have also been reported. A knockout study in mice with ERα deletion showed reduced aggression (41), whereas another study with ERβ knockout mice demonstrated an increased level of aggression (42). Additionally, female rodents showed increased aggression following ovariectomy, with the aggression levels reduced after treatment with estrogen and progesterone (43). As an estrogen-regulated gene, the loss of *Ttc9a* might disrupt estrogen activities, leading to the impaired social-related behaviors seen in this study. While TTC9A expression is induced by estrogen, it has also been shown to negatively regulate ERα activity through interacting with the co-chaperone proteins FKBP38 and FKBP51 in the ERα-Hsp90-FKPBs complex (17). A study has reported the interactive role of TTC9A and estrogen in serotonergic modulation, with estradiol (a type of estrogen hormone) administration to ovariectomized *Ttc9a*^-/-^ mice triggered anxiety and despair-like behaviors which were accompanied by the activated serotonergic system (14). Similarly, estradiol treatment down-regulated neuroplasticity-related genes in the hippocampus and amygdala of ovariectomized *Ttc9a*^-/-^ mice and resulted in increased despair-like behavior (19). Given the negative regulatory role of TTC9A in estrogen actions and the involvement of estrogen in mood regulation, it is logical to hypothesize that the alteration of TTC9A availability in *Ttc9a*^-/- ^mice contributed to the anxiogenic responses and social behavior deficits by disrupting the normal estrogen modulation. Whether the behavioral changes observed in *Ttc9a*^-/-^ animals were regulated by estrogen through the modulation on the serotonergic system and neuroplasticity, or other estrogen-related mechanisms will remain an interesting topic for future investigation. 

## Conclusion

Our study demonstrates an increase in social and non-social anxiety-like phenotypes with reduced offense responses in *Ttc9a*^-/- ^female mice. Future investigation using *Ttc9a*^-/- ^mice as the model of anxiety may promote understanding of estrogen-related mechanisms underlying social and anxiety disorders. 

## Authors’ Contributions

LWL and VCLL Conceptualized and designed the experiments. WSY, LG, SZKT, and LWL Acquired and analyzed the behavioral data. VCLL, SS, YZO, and TL, Contributed to setting up the TTC9A transgenic animals. LWL, VCLL, and WSY Drafted the manuscript. All authors reviewed and commented on the article.

## Ethical Approval

All procedures were approved by the Institutional Animal Care and Use Committee of Nanyang Technological University, Singapore(Reference number ARF-SBS/NIE-A0169 AZ).

## Availability of Data and Materials

The data that support the findings of this study are available from the corresponding author upon reasonable request.

## Funding

This work was supported by the Ministry of Education, Singapore; the A*STAR Biomedical Research Council, Singapore (06/1/221/19/455); and the Hong Kong Research Grants Council. 

## Conflicts of Interest

All authors declare that they have no competing interests.
